# The demise of large tropical brachiopods and the Mesozoic marine revolution

**DOI:** 10.1098/rsos.231630

**Published:** 2024-03-27

**Authors:** Elizabeth M. Harper, Lloyd S. Peck

**Affiliations:** ^1^ Department of Earth Sciences, University of Cambridge, Cambridge CB2 3EQ, UK; ^2^ British Antarctic Survey, High Cross, Madingley Road, Cambridge CB3 0ET, UK

**Keywords:** durophagous predation, brachiopods, grazing pressure, disturbance, refugia, Mesozoic marine revolution

## Abstract

Changes in predator–prey interactions are often implicated as drivers of major evolutionary change. A prominent example is the dramatic changes in shallow marine assemblages during the Mesozoic Marine Revolution (MMR) when major clades, including rhynchonelliform brachiopods, became restricted and less diverse. Currently, shallow-water temperate and polar brachiopods can be large, but in the tropics, they are small. By contrast, we demonstrate that throughout the Jurassic large brachiopods occurred in shallow sites, from polar to tropical latitudes, but are absent in later periods from tropical areas. These changes occurred in parallel in both major orders (Rhynchonellida and Terebratulida) and also independently within the two sub-ordinal lineages within the Terebratulida (terebratulinids and terebratellinids). Increases in both grazing and predation pressures associated with the MMR might account for this pattern. However, we note that many current environments support both large brachiopods and high densities of grazing species and suggest that the pattern fits more closely to the intensification of durophagous predation in shallow tropical waters.

## 1. Introduction

Biological interactions are major structuring factors in ecosystems, impacting organisms across scales, from morphological adaptations of individuals [1], through controlling community structure [2], to setting large-scale diversity patterns [3]. These trends, or correlations, are clear but explanation of such effects is the subject of a great deal of research in living assemblages. Profound changes in predation or grazing pressure are also often invoked to explain trends observed over evolutionary timescales, for example, the rise of biomineralization at the beginning of the Phanerozoic [4], and a wide range of morphological or life habit attributes [5,6]. Such interactions have also been invoked in an evolutionary context to explain observed shifts in clade-level dynamics such as reductions in diversity and abundance of ‘losing’ clades or their restriction into habitats that provide a refuge from attack [5,7–9]. However, despite the attraction of such hypotheses to explain changes in deep time they have proved difficult to test, as direct evidence of biological interactions in the fossil record is sparse at best, and often limited to only particular guilds of predators [5,10–12].

Brachiopods have been important marine organisms for over 500 million years. In the Palaeozoic Fauna, large rhynchonelliform brachiopods were the dominant members of most shallow marine communities [13]. In the Modern Fauna, rhynchonelliforms still occur in all oceans [14], often in dense aggregations [15–18], but they are much less diverse and widespread than in previous geological times. The decline in post-Palaeozoic brachiopod abundance and diversity has long been a topic of strong debate among evolutionary biologists [9,19,20]. Explanations often centre on either competition with bivalves [20,21], or the severity of the Permian/Triassic (PT) mass extinction [19,22]. Another hypothesis is that having been severely depleted by the PT mass extinction, brachiopods failed the challenge of the Mesozoic Marine Revolution (MMR) [9,23], either because of one or more of enhanced predation pressure [8,24–27], increased grazing impact [28–30] or loss of stability or suffocation-risk caused by increased bioturbation of the substrata [31].

The question as to whether predation, in particular, has had an important impact on post-Palaeozoic brachiopod evolution has been hotly debated by palaeontologists [21,25,26,32,33]. It is, nevertheless, clear that many modern predators (asteroids, fish, gastropods and crustaceans) do attack living brachiopods [32,34–36] and that ancient brachiopods exhibit similar shell damage patterns to those in modern assemblages [37–39]. In this paper, we seek to explore the changes in sizes of tropical brachiopods over a critical time slice of the MMR and to marry this with recent data on the impacts of crushing predators and grazing pressure on brachiopods.

One common way of assessing the impact of crushing durophagous predators uses repair frequency (RF) in populations, either from living or from fossil skeletal assemblages [5,10,37,40]. The research that led to the current study investigated RF in modern rhynchonelliform brachiopods at a global scale [41]. It revealed a distinctive pattern in shallow waters (<200 m) of highest inferred predation pressure in the mid-latitudes, with values much higher than at either polar or tropical latitudes. Although latitudinal patterns of predation, particularly in the sea, are insufficiently resolved [42], there is a generally accepted paradigm of increasing predation pressure towards the tropics [5,42–45], and there is evidence for crushing predation of molluscan prey supporting this [46]. The pattern for brachiopods, therefore, is in sharp contrast to this and, although the low RF levels at high latitudes have been explained by the paucity of durophagous predators in polar regions [47], the low RFs in the tropics, where durophagy is generally accepted to be intense [5,43–45], requires a different explanation which we explore herein by analysing size patterns in rhynchonelliforms over the course of the MMR.

There are currently no large rhynchonelliform brachiopods, over 20 mm in length, in shallow waters at low latitudes [48]. This conforms to the observation that tropical brachiopods tend to occupy cryptic refugia, such as crevices or undersides of corals [15,49]. It is traditionally noted that thecidine brachiopods are micromorphic and predominantly occur in the 10–100 m depth range in the tropics and subtropics, where they are an important part of the cryptic fauna and have been so throughout the post-Palaeozoic [26]. However, both the other extant rhynchonelliform brachiopod clades, the terebratulides and rhynchonellides, also occur in the tropics and at shallow depths where these are also characterized by small individuals [48]. This pattern has not been found in epifaunal bivalves, where taxa with large body size (e.g. *Tridacna* and *Pinctada*) may be conspicuous in shallow tropical waters [50]. These observations suggested that small size is used by rhynchonelliforms as a refuge from predation and that large size in the tropics in this clade is precluded by intense durophagous predation. If so it should be possible to look back in time for the period when durophagy caused this change in size distribution. Here, we test the hypothesis that extremely small size of modern tropical brachiopods was imposed by the increased predation of the MMR [41], by investigating the maximum size of brachiopods at different palaeolatitudes in the post-Palaeozoic record and discussing the relevance of this pattern to other alternative hypotheses, such as an increase in competition or grazing disturbance.

## 2. Material and methods

We collected data on the maximum size attained by modern brachiopod taxa by expanding upon the database created by Peck and Harper [48]. This enhanced database of 442 terebratulide and 58 rhynchonellide taxa was assembled from published accounts (70 papers and monographs) and direct measurements made from museum samples (including a large set from the wet collections of the Smithsonian Institution National Museum of Natural History (Washington, DC, USA), the Naturhistoriska riksmuseet (Stockholm, Sweden)) and our own field collections (Supplementary Information 1). The following were recorded: taxon (making no attempt to revise the generic or specific systematics), longitude and latitude of collection site, depth of collection and length of the ventral valve of the largest individual encountered (in mm). For trawl data, we used the minimum depth recorded. Taxonomic coverage of the database is good with 95% of extant genera of both terebratulides and rhynchonellides captured and including c.90% of the type species. Our database was then filtered by depth to capture sub-samples representing collection depths of less than 80 m (*n* = 122 for terebratulides (representing at least 10 129 individuals) and *n* = 12 (representing at least 1583 individuals) for rhynchonellides). We chose this depth cutoff because it coincides approximately with the photic zone depth [51], which allowed comparative material from shallow depths to be recognized in the fossil record by their co-occurrence with photosynthetic or photosymbiotic organisms (e.g. scleractinian corals and coralline algae) and also because these depths were those associated with significant shell damage [41].

Palaeontological data were collected using 170 published papers and monographs, supplemented by direct measurements of specimens in museum collections of the Sedgwick Museum of Earth Sciences (University of Cambridge, Cambridge, UK), the Oxford University Museum of Natural History (UK), the Natural History Museum (London, UK), the Naturhistoriska riksmuseet (Stockholm, Sweden) and the Smithsonian Institution National Museum of Natural History (Washington, DC, USA) (electronic supplementary material, Supplementary Information 2). For every fauna (i.e. taxa collected from the same locality and time interval) only the maximum-sized individual of the largest each of the terebratulide or rhynchonellide taxa present was recorded (i.e. data for smaller taxa were not recorded). The following data for the largest individual from each order (and for Jurassic terebratulides for each of the suborders Terebratellinida and Terebratulinida) were recorded: locality, precise age of deposit and length of the ventral valve (mm). As outlined above, only those samples collected with clear evidence of occurrence in the photic zone were included. Palaeolatitudes were derived either using the established value on the Paleobiology Database (https://paleobiodb.org) or from published local palaeogeographic reconstructions.

In our analysis, the principal comparison is between the Modern and Jurassic brachiopods. The Jurassic period was chosen because it directly pre-dated the second major MMR pulse [52].

## 3. Results

**Figure 1.**
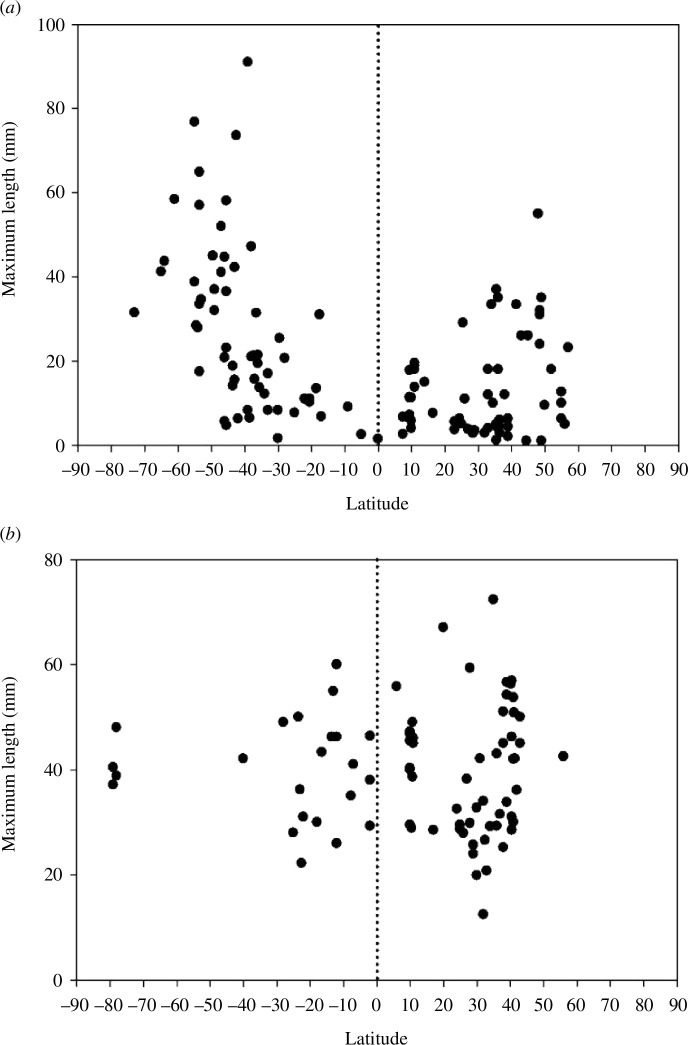
Distribution of lengths of shallow (<80 m water depth) terebratulide brachiopods plotted against latitude during life. (*a*) Extant taxa, each point records the length of the largest individual of a particular taxon at a given locality. Latitude versus maximum size of recent terebratulides <80 m water depth (*n* = 122). (*b*) Jurassic taxa, each point records the maximum length of any terebratulide brachiopod collected at a particular sampling site plotted against the palaeolatitude of that site. Palaeolatitude versus maximum size of Jurassic terebratulides in shallow water (*n* = 78).

**Figure 2.**
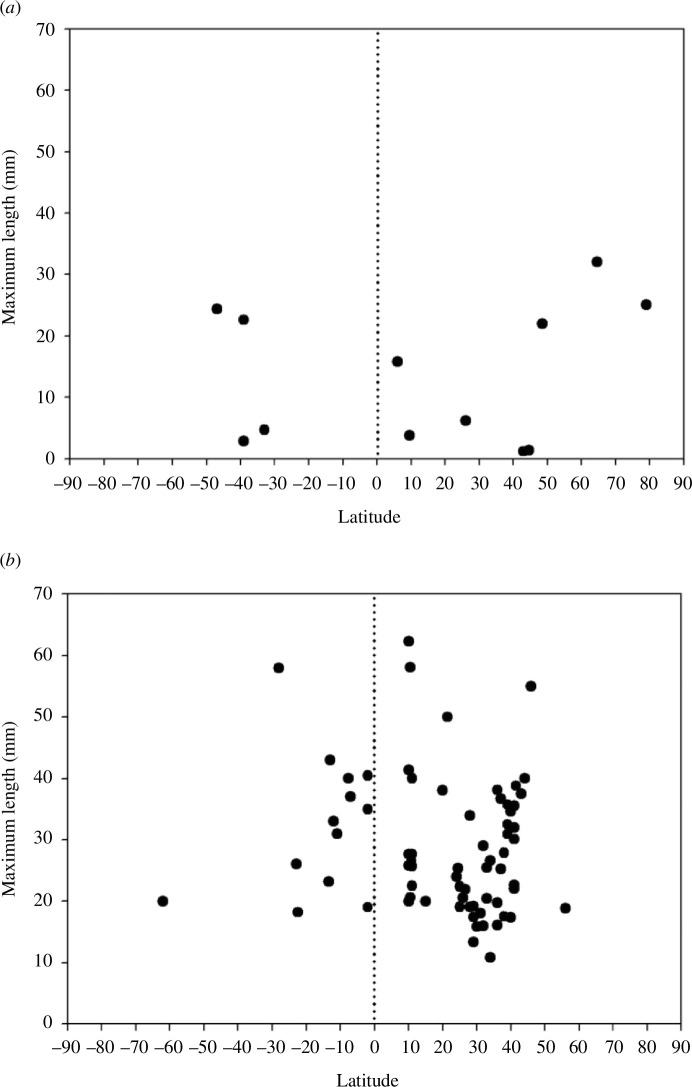
Distribution of lengths of shallow (<80 m water depth) rhynchonellide brachiopods plotted against latitude during life. (*a*) Extant taxa, each point records the length of the largest individual of a particular taxon at a given locality. (*b*) Jurassic taxa, each point records the maximum length of any rhynchonellide brachiopod collected at a particular sampling site plotted against the palaeolatitude of that site.

Global size trends with latitude for modern or palaeolatitude for Jurassic taxa are strikingly different for both terebratulides and rhynchonellides (figures 1 and 2). Our expanded and more focused database reveals a pattern for recent shallow-water terebratulide taxa (*n* = 122) like that of Peck and Harper [48] (figure 1*a*); this most diverse modern brachiopod clade often exceeds 50 mm length, and the largest species occur in the mid-latitudes (30°−60°) in both hemispheres (the largest being the Chilean *Magellania venosa* which exceeds 90 mm length). Up to 10° either side of the equator, all records are uniformly small and even up to 25° latitude the maximum size is only 26.6 mm, apart from *Kraussina rubra* from Namibia, which inhabits the atypical rich cold upwelling Benguela Current [53]. The difference in size between the largest tropical species (between 20˚N and 20˚S) and those at higher latitudes is significant, as shown by a comparison of the 20% largest species in both groups (Mann–Whitney *U* statistic = 0.000, *n* = 4,10, *p* = 0.002). Larger tropical taxa than those shown in figure 1 do occur, but only in deeper waters (see electronic supplementary material, figure S1), for example, the largest terebratulide record in the tropics is 57.6 mm long from beyond 800 m depth off Jamaica.

By contrast, shallow-water Jurassic terebratulides have a very different size distribution (figure 1*b*), with large individuals occurring throughout the low latitudes (in 30 records from the palaeotropics the maximum length recorded was 67 mm) and there is no discernible latitudinal trend in maximum size. When the same analysis as that done comparing the size of the largest tropical species with lower latitudes in living species is done for Jurassic terebratulides there is no significant difference (Mann–Whitney *U* statistic = 53, *n* = 6,11, *p* = 0.93). Terebratulides have two sub-ordinal lineages (the terebratulinids and terebratellinids) which had separated by the end of the Triassic [54]. Interestingly, both of these include large shallow-water tropical Jurassic taxa and both undergo parallel trends of size reduction in shallow tropical water across the MMR (see Supplementary Information 2). Additionally, we note that the only Jurassic superfamily with extant representatives—the Zeillerioidea—contains large taxa, but is today found only in deeper bathyal or even abyssal waters [54].

Data for modern rhynchonellides are more limited, as they are much less diverse, particularly in the tropics [55], and because they predominantly occur in deeper water today [26,56,57]. Modern rhynchonellides attain smaller maximum sizes than terebratulides (the largest reported is 32 mm long). There are few records from water depths shallower than 80 m and only two from tropical sites, the largest of which is 15.8 mm (figure 2*a*), but rhynchonellides up to 30 mm length do occur in deeper tropical waters (electronic supplementary material, figure S1). By contrast, there were far more shallow-water rhynchonellides in the Jurassic [55,57]. Our data show Jurassic shallow-water rhynchonellides with large taxa (up to 62.3 mm) present across all latitudes, and the largest tropical Jurassic species was 58.1 mm long (figure 2*b*).

Having established that the modern size distribution of shallow-water brachiopods is strikingly different from that of the Jurassic, it is of interest to establish when the pattern changed. Although palaeotropical data for brachiopods are much sparser in the later periods than for the Jurassic itself [58], there are no records of large taxa in this region at any time since the Jurassic (electronic supplementary material, figures S2 and S3) except for records for the Caribbean during the Paleocene, which are reported to be, like those from modern Namibia, influenced by a cold upwelling [59].

## 4. Discussion

Our data indicate that, although large brachiopods were present in the shallow-water palaeotropics in the Jurassic, large taxa were, and remain to the present day, excluded from these habitats after this period. This pattern of size distribution is evident in all the extant brachiopod clades; terebratulides (including the two suborders that had already separated prior to the Jurassic [54]), rhynchonellides and thecidines.

The MMR is a complex multifactor event, and the relative importance of competition, grazing and predation are difficult to unravel. However, information on the likely importance of the main factors involved can be obtained from the analysis of the ecology of living brachiopod species. The size distribution of modern shallow-water brachiopods in the tropics is generally below the minimum size at which repaired damage has been recorded in populations living at higher latitudes [41]. However, larger brachiopod taxa do occur at these latitudes but only in deeper waters where durophagous predators are much less active, as shown by the low observed repair frequencies beyond 100 m [41].

Small body size has two advantages in avoidance of predation. Firstly, it facilitates, but does not necessitate, occupation of cryptic habitats such as crevices or the undersides of reef-building organisms. These confined habitats offer protection from larger grazers and predators. Secondly, low flesh yield reduces prey value [60], and the data for brachiopods demonstrate size refuges for both individuals below a threshold size and for large individuals above a critical size [61]. Clearly, small size does not provide immunity from predation; predatory drill holes occur in a range of micromorphic brachiopods [32,62,63] and grazing species such as sea urchins consume small species, including brachiopods [15,28]. Other potential defensive adaptations in shallow-water brachiopods include cryptic colour patterning [64] and ornamentation, noting also that spiny terebratulides were not present, or were rare before the MMR, but much more common after [27], another factor indicating an effect of increased predation.

Aside from predation pressure, other biological interactions intensified during the MMR [5,7] and might be considered as potential causes of the changes described here. However, although we accept that competition and grazing may be locally important, we reject their major roles here. In a seminal work, Stanley [8] concluded that competition rarely, if ever, results in the removal of species, and that predation is a far stronger factor in species elimination than competition. In ecology, the competitive exclusion principle, often called Gause’s law [65], states that when two species compete for the same resource one must be driven to extinction by competition. However, such exclusion is very rare in ecology arguing against significant competitive exclusion [66]. Modern shallow-water brachiopod communities at all latitudes coexist with a wide range of sessile encrusters, such as sponges, ascidians and bryozoans, often in dense aggregations [18,67 and see electronic supplementary material, figure S4], and all of these taxa are competing directly for space. To argue that increased competition was important in restricting the size of shallow-water tropical brachiopods would require evidence of a decrease in competition between encrusting communities with increasing latitude. Although competition has been shown to decrease with latitude in encrusting Bryozoa [68], there is still intense seabed competition in Antarctica. Competition in bryozoans also becomes more polarized towards the poles, with poorer competitors failing more frequently at high latitude [69]. Furthermore, these studies on bryozoans have all been conducted at shallow depths, <30 m where iceberg scour and anchor ice have strong effects and exclude brachiopods [70]. At depths beyond 30 m, however, the Antarctic seabed is characterized by very dense and abundant communities dominated by encrusting sponges, holothurians and ascidians [67 and electronic supplementary material, figure S4*c*]. The biomass of these communities is around an order of magnitude higher than similar areas of seabed in boreal and subtropical regions [70,71]. Such a high standing biomass and permanent standing stock indicate that spatial competition in Antarctica is intense, and the existence of large brachiopods (largest recorded is 58.4 mm) there, suggests that size is not restricted by competitive interactions.

Increased grazing is the other potential factor to explain the exclusion of large tropical brachiopods since Jurassic times. However, there are several lines of information arguing against this. Firstly, by the Jurassic grazing of hard substrata was already well-developed [29,72,73]. Secondly, there are modern shallow-water brachiopod communities at middle and high latitudes where large taxa coexist with strong grazing pressures (see electronic supplementary material, figure S4). For example, the brachiopod fauna of Antarctica is among the most diverse in the current oceans with 63 species recorded there [74], some of which are large and exceed 50 mm in length [60]. The Antarctic seabed has also been reported to have extremely high densities of grazing species, with the urchin *Sterechinus neumayeri* occurring at densities up to 600 m^−2^ and a biomass of over 500 g m^−2^, and the limpet *Nacella concinna* regularly has densities reported over 200 m^−2^ [75–77]. These are as high or higher densities of grazing species reported in Antarctic as anywhere globally. We found no reports of similarly high grazer densities in the tropics, where mean densities of 7.7 m^−2^ and highs of 25 m^−2^ were reported for the Caribbean from a compilation of over 70 published sources [78], and such densities are considered very high for coral reefs in general [79]. Similar surveys in the Seychelles found mean urchin densities of 0.02 m^−2^ and maxima of 0.16 m^−2^ [80], in Kenya mean densities were less than 10 m^−2^ and the maximum was between 50 and 60 m^−2^ [81], in the Florida Keys these values were 0.3 and 0.6 m^−2^ [82], and in Moorea, urchins occurred at densities less than 0.03 m^−2^ [83]. A further consideration is that small individuals or taxa ought to be particularly susceptible to grazing [30,84], with larger individuals reaching a size refuge. Such a solution would favour rapid growth and should be easier to attain in the tropics where growth rates in the oceans are fastest at lower latitudes, and an order of magnitude faster in tropical bivalve molluscs compared to polar species [85] while the brachiopod *Liothyrella neozelanica* in New Zealand grows 3.3–5.4 times faster than the Antarctic congeneric, *Liothyrella uva* [86].

In conclusion, our data show that large brachiopods were lost from habitats where durophagous predation is most intense in tropical shallow areas, and this pattern was established during the early part of the MMR and persists to the present day. The current data do not allow us to determine whether the decrease in size in tropical brachiopods was due to either exclusion of large, and the survival of small taxa or to miniaturization of existing specific lineages by paedomorphic processes.

## Data Availability

Data are available in the electronic supplementary material [87].
